# Vitamin E and Clonazepam in the treatment of tardive dyskinesia secondary to atypical antipsychotic treatment: a case report

**DOI:** 10.1192/j.eurpsy.2025.2103

**Published:** 2025-08-26

**Authors:** S. Uygun, H. Kaya, E. Göka

**Affiliations:** 1 Department of Psychiatry; 2 Ankara Bilkent City Hospital, Ankara, Türkiye

## Abstract

**Introduction:**

Tardive dyskinesia is a movement disorder mostly associated with long-term antipsychotic use. Patients may present with a wide variety of clinical features such as stereotypic movements, dystonia, etc. There are many treatment approaches but the level of evidence is low. (Vasan et al. StatPearls Pub 2024)

**Objectives:**

To draw attention to the clinical presentation of tardive dyskinesia and its treatment.

**Methods:**

A detailed case report is documented.

**Results:**

A 43-year-old woman, married, housewife, diagnosed with psychotic disorder. Last year, she admitted to our outpatient clinic for the first time. She was receiving Amisulpride 400 mg/g, Zuclopentixol deconoate 200 mg/month, Biperiden 4 mg/g, Propranolol 80 mg/g on admission. Her complaints were slowed movements and difficulty in doing housework. On examination, mouth puckering and periodic head movement were noticeable. She complained of contractions in her legs and numbness in her tongue. Her speech and walking were slow. In cerebellar tests she was clumsy. She stated that her complaints increased sometimes, the contraction in her neck and legs never stopped, but the mouth puckering movement ceased at night. Her AIMS scale point was 11.

It was learned that about 4 years ago she had started to complain of feeling that she was being spied on and hearing bad things. She had been hospitalized and diagnosed with psychotic disorder. Paliperidone and aripiprazole long-acting injections, olanzapine, quetiapine treatments had been tried during hospitalization and outpatient follow-up. She had been continuously switched to another treatment because of her suspicion. Lastly zuclopentixol deconoate had been started and combined with amisulpride. She stated all her complaints of movement appeared shortly after first hospitalization. Biperiden and propranolol were added to treatment, but her complaints didn’t improve.

During our follow-up she was consulted to neurology. Her current picture was evaluated as tardive dyskinesia. Her treatments were gradually stopped. Clonazepam was started and gradually increased to 2 mg/g and combined with vitamin E and its dosage was increased to 1200 mg/g. After discontinuation of antipsychotics during close follow-ups she had no psychotic complaints. Within 5 months, mouth puckering movement, stereotypic left-turning movement in the neck, numbness in the tongue and contraction in the leg completely resolved. Her speech and walking improved. Last AIMS scale point was 3.

**Conclusions:**

Tardive dyskinesia is an iatrogenic movement disorder. Many methods have been proposed for treatment but no definite treatment is known. It’s thought that vitamin E may be beneficial with its antioxidant effect and clonazepam may be effective above 2 mg. The case is important in terms of demonstrating the efficacy of the combination of vitamin E and clonazepam. (Cornett et al. *Ochsner journal* (2017):162-174.)

**Disclosure of Interest:**

None Declared

